# Review Article: Current Concepts in the Treatment of Congenital Clubfoot

**DOI:** 10.1055/s-0044-1787769

**Published:** 2024-12-21

**Authors:** Francisco Nogueira, Pedro Poggiali

**Affiliations:** 1 Rede Mater Dei de Saúde, Belo Horizonte, MG, Brasil

**Keywords:** clubfoot/diagnosis, clubfoot/etiology, clubfoot/surgery, manipulation orthopedic, orthopedic procedures

## Abstract

Congenital clubfoot is a complex, frequent deformity that can be challenging even in experienced hands. The Ponseti method remains universally accepted as the gold standard for treatment, and excellent outcomes are within expectations in most cases with appropriate technical management. Recurrences continue to be a problem and are mainly associated with non-compliance with orthosis use. However, other factors may influence the risk of recurrence and contribute to an unsatisfactory outcome. Tibialis anterior transfer balances deforming forces and helps correction as long as the foot is flexible. A recurred deformity is not corrected spontaneously, it requires treatment. Adjuvant surgical procedures should be part of the orthopedist's therapeutic arsenal.

## Introduction

Congenital clubfoot (CC) is one of the most common congenital musculoskeletal disorders. It consists of rigidity of the deformity with equinus, varus, cavus, and adductus components. Its incidence ranges from 0.39 to 7 for every one thousand live births according to ethnicity, and it is more common in males and bilateral in 50% of cases. 1 2

The etiology is multifactorial, and its most common form is idiopathic. However, CC is also frequently associated with neuromuscular diseases, such as myelomeningocele, arthrogryposis, and several syndromes. 1 2 3

Maternal smoking, gestational diabetes, maternal age, and viral infections are risk factors for CC. Many genes have been linked to idiopathic CC, with a hereditary component of up to 20%. 3

Rigidity differentiates CC from postural deformities. It is easy to identify this rigidity during the first physical examination when complete correction by foot mobilizing is impossible.

Although CC has a spectrum of higher or lower severity, it presents the following abnormalities: contracture of the Achilles tendon, deltoid and spring ligaments shortening; posterior tibial tendon shortening; and tension of the posteromedial capsuloligamentous structures. These abnormalities lead to medial deviation of the navicular cuboid and subtalar varus, generating a deformity in inversion, adduction, and consequent supination. The cavum is secondary to the bending of the first ray.

Ideal treatment must correct these deformities, resulting in a plantigrade, flexible, functional, and painless foot. 1 2 The objective of this article was to review current concepts in idiopathic CC management.

## History

The first congenital clubfoot (CC) records date back to ancient Egypt, in the twelfth century BCE. In 400 BCE, Hippocrates described the method of manipulation and immobilization for CC treatment, followed by using special shoes to maintain the correction. Tenotomy development occurred in the eighteenth century. New surgical techniques emerged in the nineteenth century with the introduction of anesthesia and antisepsis. 1 2

From then on, surgeries with an extensive release of soft tissues, such as those described by Turco, McKay, and Simons, prevailed. However, at the same time, less invasive treatments gained momentum.

In 1939, Kite described his technique of manipulation and serial plastering. However, the correction fulcrum in the calcaneocuboid bone limited varus correction and required soft-tissue release in 50 to 75% of cases. 1 2

In the decade following Kite's publication, Ponseti developed his method at the University of Iowa Medical School. Studying several CC correction techniques, Ponseti observed that most required surgical approaches, resulting in stiffness, weakness, and extensive scarring.

**Fig. 1:**
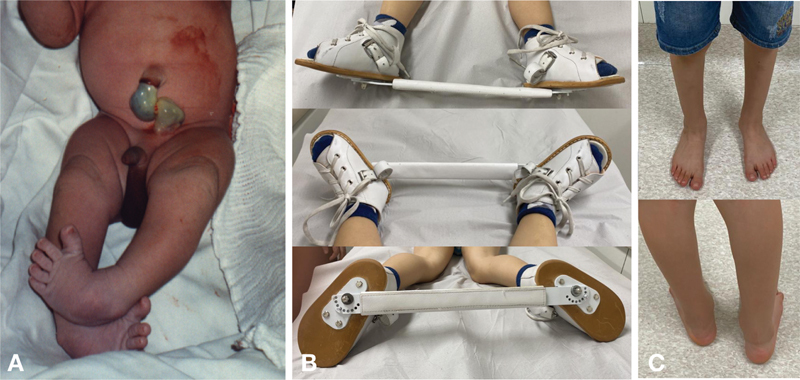
( **A** ) Newborn with bilateral congenital clubfoot (CC). ( **B** ) A 3-year-old child with right-sided unilateral CC using an abduction orthosis (Denis Browne) for 14 hours per day. Note how the affected (right) foot (right) must be at 70° of abduction while the left foot is at 40°. ( **C** ) A 7-year-old patient showing the therapeutic outcome of the Ponseti technique for a right-sided CC.

Observing Kite, then the leading advocate for conservative treatment, Ponseti concluded that the failure resulted from a misunderstanding of the anatomy and biomechanics in CC. He concluded that by using the fulcrum on the talus and putting pressure on the first metatarsal, supinating the foot, he could unlock the subtalar, an essential step at the initial manipulation. This realization started a revolutionary method. In 1963, Ponseti published his first long-term outcomes and, today, the Ponseti technique is the gold standard for CC treatment ( Fig. 1 ). 1 2

### Assessment and Classification

There are four CC types: postural, idiopathic, neurogenic, and syndromic. Postural CC is flexible and evolves with spontaneous correction. Mobilization can help achieve a faster correction. A CC requiring manipulation and immobilization is probably less severe. 2

Idiopathic CC has no known cause, as the name suggests. Its main feature is rigidity and requires treatment with manipulation and plaster immobilization. 2 4 Achilles tendon tenotomy is necessary in most cases, and maintaining correction with orthosis is essential to prevent recurrence.

Idiopathic CC is a broad group of conditions with a wide severity range and greater or lesser rigidity. Despite the success of the Ponseti method in treating most idiopathic CC cases, some feet are more resistant. Sometimes, they may require more complex surgical approaches and present more recurrences. It is believed that muscular and genetic components determine this higher resistance. 4

Syndromic and neurogenic types can also respond to the Ponseti method despite being more rigid, resistant, and presenting a higher recurrence rate. 5

Different classification systems evaluate CC severity, prognosis, and therapeutic outcome. The two most used classifications are those from Pirani and Diméglio. 2 6 7 8 9

Diméglio's CC classification considers four main parameters: equinus, varus, rotation, and adduction, each receiving a score from one to four according to reducibility. Secondary parameters, when present, add up to one point each: posterior fold, medial fold, cavus, and muscular condition (triceps surae or tibialis anterior contracture or hypertonia, peroneal muscle weakness, or both). Thus, each foot receives a score from 0 to 20, dividing the condition into 4 types according to the sum: mild, moderate, severe, and very severe CC. 6

Grade I (0–5 points): mild, flexible, and benign CC, similar to postural clubfoot. Diméglio even suggested its exclusion from statistics for positively distorting the results.
Grade II (5–10 points): moderate, reducible, but partially resistant CC.
Grade III (10–15 points): severe, resistant, but partially reducible CC. Diméglio reported it as the most common type.
Grade IV (15–20): very severe CC. The foot is very rigid, virtually irreducible, and a type of pseudoarthrogrypotic foot.

Pirani's classification relies on six parameters, three related to the hindfoot (posterior crease, calcaneus palpation, and equinus reducibility) and three related to the midfoot (medial crease, talus coverage, and lateral edge of the foot). Each of these items receives a score from 0 (no abnormality), 0.5 (abnormal), to 1 (very abnormal), reaching a total of up to 6 points. It is a useful scale for evaluating treatment progress. 2 9

Both classifications have documentation value but little practical use. Despite good reproducibility, their usefulness for predicting prognosis and guiding treatment is controversial. However, recent studies suggested that both may help predict the number of casts and contribute to a better expectation alignment with the family. 2 9

### Treatment

After CC diagnosis, treatment must start early. The prenatal consultation is an opportunity to guide parents about the natural history, expectations, prognosis, and treatment strategies. From the first contact, one must emphasize that family support throughout the process is essential for the desired outcome.

Ponseti suggested starting treatment in the first week of life to take advantage of the neonatal higher flexibility. 2 However, this is not an orthopedic emergency. Zionts et al. 10 found no differences in cast numbers, skin lesion incidence, treatment adherence, or recurrence when comparing the age at the start of treatment in 176 patients (median of 4 weeks old). We suggest assessing the newborn individually, considering other factors to determine the beginning of treatment. It is reasonable to wait for the first few days of family adaptation, the first consultation with the pediatrician after discharge, the establishment of breastfeeding, and confirmation of effective weight gain. Once conditions are favorable, one must not delay treatment.

**Fig. 2:**
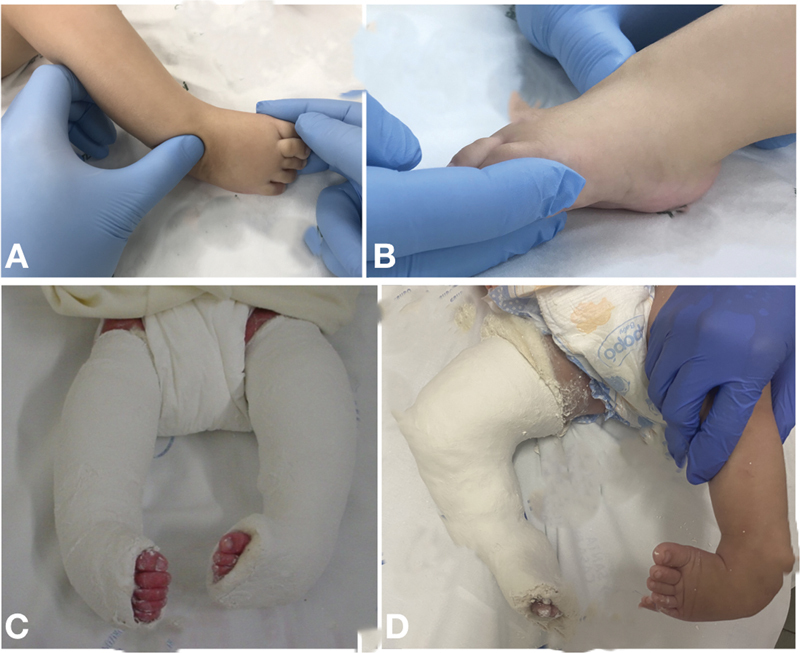
Ponseti technique: ( **A,B** ) Detail of manipulation with the fulcrum on the head of the talus and abduction maneuver with the foot in supination. ( **C,D** ) Examples of the first cast for cavus correction with the foot in supination.

The Ponseti method is the first choice, including in cases of non-idiopathic CC. 1 2 11 It consists of manipulation and immobilization with a weekly inguinopodal cast to lengthen posteromedial structures with contractures and gradually correct deformities. The fulcrum of manipulation must be on the lateral surface of the head of the talus, which is palpable on the lateral dorsum of the midfoot ( Fig. 2 ). The first deformity for correction is the cavus, with support on the neck of the first metatarsal and a supination maneuver of the forefoot, to place it in adequate alignment with the rearfoot. The mistaken perception that pronation is necessary to correct the foot enlarges the cavus, leading to an iatrogenic deformity. 1 2

Next, adductus, varus, and equinus corrections begin progressively through an abduction maneuver. Counterpressure from the thumb on the head of the talus prevents its rotation in the ankle clamp. Varus, inversion, and rearfoot adduction correction are simultaneous, as the tarsal joints have a strict mechanical interdependence and cannot sustain correction in isolation. 2

A well-molded cast is essential to keep the foot in the correct position and prevent the cast from slipping. Use only a thin layer of cotton and manipulate gently without forcing the correction. Avoid continuous pressure on the talus during cast creation and cast well over the posterior tuberosity of the calcaneus and around the malleoli. Do not touch the calcaneus during manipulation or casting to avoid limiting subtalar mobility and compromising varus correction. Molding is a dynamic process, so constantly move your fingers to prevent excessive pressure on any bony protrusion, and continue molding until the plaster hardens. 1 2 Heated water speeds up plaster drying. Initially, make the plaster boot cover the toes and then finish it, exposing the toes but maintaining their plantar support. Next, extend the plaster to the root of the thigh, always keeping the knee at 90° of flexion. 2 12 Extending the knee can facilitate the passage of the plaster, especially in smaller babies, but avoid it so that there is no pressure on the popliteal region of the thigh after knee flexion.

**Fig. 3:**
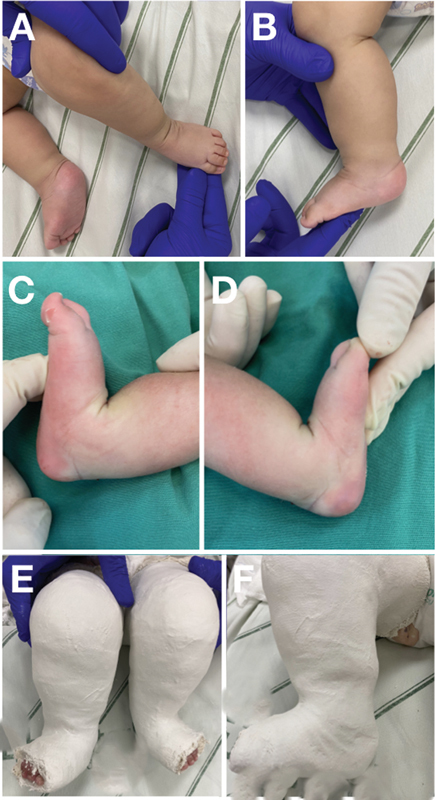
( **A,B** ) Congenital clubfoot partially corrected after four Ponseti casts sustaining limited dorsiflexion. ( **C,D** ) Immediate outcome after percutaneous tenotomy with satisfactory gain in dorsiflexion. ( **E,F** ) Detail of the post-tenotomy cast, maintaining abduction and dorsiflexion.

On average, equinus correction requires four to six plaster changes. Equinus deformity is the last one corrected. Tenotomy, required in approximately 90% of cases, must occur after cavus, adduct, and varus component correction, with foot abduction of at least 50° concerning the tibial plane ( Fig. 3 ). Equinus correction with forced manipulation can lead to a inkblot deformity, because, unlike the tarsal ligaments, which can undergo stretching, the Achilles tendon consists of thick, non-distensible collagen fibers with few cells. 2 However, in some cases, the dorsiflexion gain is simultaneous to the correction of other deformities. When this happens, and the dorsiflexion is at least 15°, tenotomy is unnecessary. 1 2 13

Ponseti recommended performing tenotomy percutaneously in an outpatient setting, especially due to the lower cost, easy reproduction, and no anesthetic risk. 2 14 However, considering the low risk of current anesthesia, the ease of performing the procedure with the anesthetized patient, and the better management of eventual bleeding in a more controlled environment, many surgeons choose to do it under anesthesia. 15 Take care not to leave the tenotomy incomplete during the percutaneous approach, resulting in an unsatisfactory correction. Performing the procedure under direct visualization of the tendon through small approaches is recommended, particularly in cases of a new tenotomy after recurrence. 13 In patients with myelomeningocele, resection of approximately 1 cm of the Achilles tendon is recommended to reduce the recurrence risk, as demonstrated by Arkin et al. 5

The last cast must be created with the foot in hypercorrection, with around 70° of abduction and 20° of dorsiflexion, and kept for 3 weeks. Then, the abduction orthosis begins to be used for 23 hours during the first 3 months and then for 14 to 16 hours a day until the age 4. 1 2 16

The abduction orthosis must maintain the corrected foot at 70° of abduction and, in patients with unilateral CC, the unaffected foot must be at 40° of abduction ( Fig. 1 ). The bar should be shoulder-width apart and have a curvature of around 10 degrees with the convexity downwards, helping to keep the feet in valgus and dorsiflexion. It is essential to keep the foot well-positioned and fully supported, with tight straps and laces to prevent the heel from rising and an equinus deformity development.

The success rate of idiopathic CC using the Ponseti method is higher than 90%, as long as the technique application is correct and orthosis adherence continues until the recommended age. 2 16 A frequent concern for parents is whether prolonged treatment will cause some delay in the child's development, but this is not relevant. A minimum delay (1.5 months) to reach the main milestones of gross motor development, and an average delay of 2 months for independent walking are within expectations. 17 The ability to practice sports also does not seem impaired in school-aged children undergoing proper treatment. 18

#### Complex CC

**Fig. 4:**
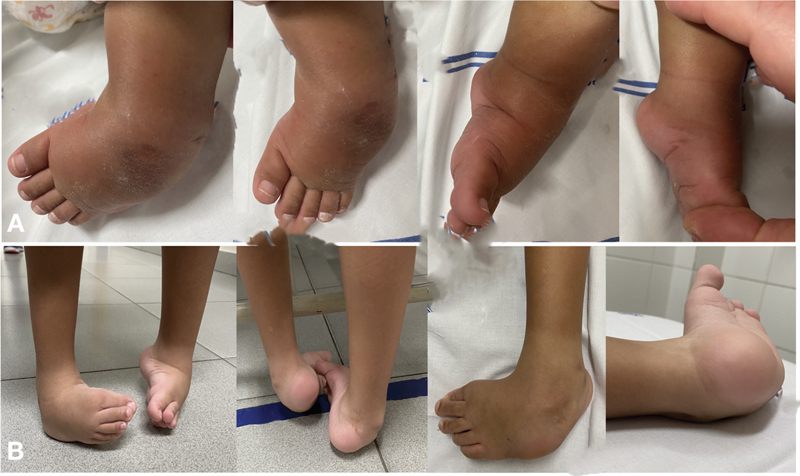
( **A** ) Example of a congenital clubfoot (CC) patient presenting rigidity, shortening of the first ray, hallux extension, and plantar transverse crease. ( **B** ) Example of neglected CC in a 3-year-old patient.

Ponseti described complex CC as a variation of idiopathic CC. Complex CC consists of a rigid equinus deformity, severe plantar flexion of the metatarsals, a deep crease above the heel, a transverse plantar crease, and shortening of the first metatarsal with hallux hyperextension ( Fig. 4 ). These characteristics are not evident at birth, but present during treatment and may be related to an inadequate technique. Their identification usually follows plaster slippage. 19 20

Complex CC requires some care in its management and must be properly recognized. Although forefoot adduction is easily corrected, the metatarsals remain in plantar flexion with the calcaneus fixed in an equinus deformity, and attempts to correct the remaining deformities by persisting with forefoot abduction maneuver lead to metatarsal hyperabduction. Ponseti recommended performing the correction with a fulcrum on the head of the talus and the lateral malleolus. Do not abduct the forefoot beyond its normal alignment. After varus correction, forefoot flexion and equinus deformity corrections are simultaneous by dorsiflexing the metatarsals with both thumbs. However, tenotomy must be performed to correct the residual equinus deformity, with care to avoid metatarsal hyperextension and consequent progression to an inkblot deformity. To prevent the cast from slipping, immobilize the knee in at least 110° of flexion. 2 19 20

#### Recurrence

Recurrence of any CC component (equinus, cavus, varus, adduct, and supination) in a previously corrected foot constitutes a recurrence. Dorsiflexion loss is usually the first observed abnormality. A dorsiflexion of at least 20° up to age 3 and 15° up to age 5 in the corrected foot is within expectations. 21 22 Rieger and Dobbs considered a dorsiflexion lower than 15° as a recurrence. 1 Dietz suggested that recurrence treatment should start with less than 10° of dorsiflexion. 22 For unilateral CC, a difference of 10° between the feet is expected, and an increase in this difference may indicate a recurrence. 22

A recurred deformity is not corrected spontaneously, it requires treatment. A short period of manipulation and weekly casting followed by resuming brace use is usually enough. A new tenotomy may be necessary to obtain satisfactory dorsiflexion. 2

Supination observation occurs after gait starts with dynamic varus and supination, support on the lateral edge, and thickening of the lateral plantar skin. This phenomenon occurs due to the imbalance between the strong tibialis anterior muscle and the weak peroneal muscles. These cases may require anterior tibial tendon transfer (ATTT). Anterior tibial tendon transfer is usually indicated from 3 years old, after lateral cuneiform ossification. Its performance must occur only when the deformity is dynamic and the foot is flexible, that is, feasible to complete correction with passive mobilization. Any rigid deformity correction must use plaster changes, a potential tenotomy of the Achilles tendon for equinus deformity correction, and even osteotomies, when necessary, before ATTT. 2 13

It is worth highlighting that the most common cause of recurrence is non-compliance with the use of the orthosis. Recurrence is about ten times greater when this occurs. According to Desai et al., recurrences occur in only 6% of cases properly following the protocol and in more than 80% if there is no adherence. 1 2 16 Zionts et al. found a 42% risk of needing an ATTT in patients who did not follow the protocol of orthosis use compared to 19% for patients properly using orthosis. 23

Adherence to orthosis use is a better predictor of recurrence than the severity of the deformity at birth. 1 16 However, recurrence still occurs despite the correct use of the orthosis, suggesting the involvement of other components not fully elucidated. Although the etiology is multifactorial and not yet completely understood, increasing evidence points to a neuromuscular etiology and consequent deforming forces persisting after the initial correction. 2 23 24

When recurrence occurs despite proper orthosis use, there is likely a more relevant neuromuscular component and higher peroneal weakness. Anterior tibial tendon transfercorrects this imbalance and improves biomechanical parameters, making it unnecessary, in most cases, to use an abduction orthosis after the procedure. 13 23 24 25 Little et al. studied 104 patients (172 feet) and showed that evertor muscle weakness is a fundamental recurrence cause. In patients with weakness, 67.9% had a recurrence, and 50% required additional surgery; on the other hand, none of the patients with good evertor strength had a recurrence. 24

### Clubfoot After Starting to Walk

A neglected CC is a CC with no prior treatment in patients who started to walk ( Fig. 4 ). A neglected CC requires differentiation from a residual CC, that is, a CC with no complete correction, and a recurrent CC. Regardless of age or severity, the Ponseti method is the choice for treating neglected, residual, or recurrent CC. 26 27

In neglected CC, the Ponseti method can result in surprising outcomes even in older patients. However, it requires more plaster changes, and the likelihood of additional surgery is higher. 13 26 27 Lourenço and Morcuende 26 recommended manipulating these feet for 5 to 10 minutes before plaster immobilization and keeping the plaster for 2 weeks for higher flexibility gains.

Even in cases of late recurrence or residual deformity in the resistant foot, preoperative plaster changes contribute to gaining flexibility and potentially bring some degree of correction, reducing the extent of the surgical intervention. 13 26 27

**Fig. 5:**
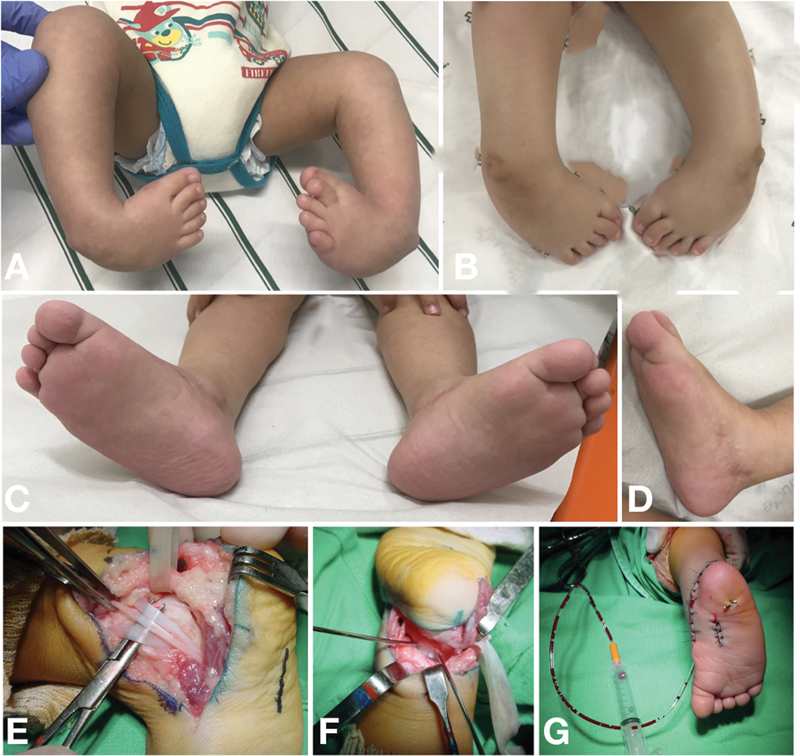
( **A** ) Newborn with congenital clubfoot with arthrogryposis; initial appearance. ( **B** ) After 12 weeks of manipulation using the Ponseti technique, the feet remained rigid and resistant. ( **C,D** ) Result at 2 years old, after posteromediolateral release. ( **E–G** ) Details of the radical release technique through the Cincinnati incision. ( **E** ) Isolated neurovascular bundle and tibialis posterior, flexor digitorum longus, and flexor hallucis longus tendons. ( **F** ) Talus derotation maneuver, as described by Hsu et al. 29 ( **G** ) Outcome after fixation of the subtalar and talonavicular joints.

Although numerous techniques have been described for CC treatment, one must currently consider adjuvant procedures in case of correction failure using the Ponseti method 4 13 ( Fig. 5 ).

The main surgeries are soft tissue and bone procedures. Historically, wide-release techniques, posteromedial or posteromediolateral, are effective for correcting deformities and achieving a plantigrade foot. However, they lead to fibrosis, scar retraction, and consequent stiffness, with unsatisfactory long-term outcomes. 28 29 Wide releases should be preferably used in non-idiopathic cases. When indicated as an adjuvant treatment to the Ponseti method, it must be tailored, using the smallest possible approach to correct only deformities that did not respond to manipulations and plaster changes. 13

If there is no equinus deformity improvement after tenotomy, one must perform a posterior release (tibiotarsal and subtalar capsulotomy). In this case, careful dissection is recommended to identify the peroneal tendons, flexor hallucis longus, flexor digitorum longus, and the neurovascular bundle ( Fig. 5 ). After identifying these structures, perform a posterior capsulotomy starting laterally from the sheath of the peroneal tendons, and going medially to the sheath of the flexor hallucis longus. 13

If required, one can extend the posterior release medially, laterally, or both for complete peritalar release, but with the disadvantages and complications already described. Always spare the deep deltoid to avoid valgus overcorrection. However, talocalcaneal interosseous ligament resection is controversial. 13 30

In cases requiring posteromediolateral release, Hsu et al. recommended talus derotation aided by a Kirschner wire acting as a joystick to optimize the correction before talonavicular and subtalar fixation 29 ( Fig. 5 ).

Other potentially required procedures include calcaneocuboid capsulotomy, fasciotomy, medial column joint plantar capsule release, digital flexor, hallucis flexor, and tibialis posterior muscle stretching, and hallux abductor tenotomy.

Bone procedures are reserved for older patients with non-reducible, structured deformities. Heel varus correction can be accomplished with the Dwyer technique (lateral wedge resection osteotomy). An opening wedge in the medial cuneiform can be associated with a lateral closing wedge in the cuboid to optimize adduct correction. An osteotomy with a dorsally based wedge in the midfoot helps correct the cavus. Anterior hemiepiphysiodesis of the distal tibia is an option for residual and refractory equinus deformity. Salvage procedures, such as naviculectomy combined with lateral calcaneocuboid shortening, as described by Mubarak, talectomy, and triple arthrodesis, should be reserved for severe cases and older patients, as well as the use of circular external fixator with gradual distraction and correction in multiple plans. 4 13

## Final Considerations

The Ponseti method remains the gold standard for CC treatment. It is critical to identify the complex foot as its proper management requires a change in technique. Recurrence remains an issue, and its main cause is non-adherence to the abduction orthosis use protocol. Anterior tibialis transfer balances the deforming forces in the recurrent and dynamically supinated foot as long as it is flexible. Residual or recurrent deformity is not corrected spontaneously, and no tendon transfer can correct a foot that is not passively correctable. Adjuvant surgical procedures must be part of the therapeutic arsenal of the orthopedist for CC treatment.
