# Supplementary material for: Review Article: Current Concepts in the Treatment of Congenital Clubfoot
Source: Rev Bras Ortop (Sao Paulo). 2024 Dec 21;59(6):e821–9. doi: 10.1055/s-0044-1787769 (PMC11663064; doi:10.1055/s-0044-1787769)
Supplement: Related texts

# Research article: Artigo de revisão: Conceitos atuais no tratamento do pé torto congênito

**Keywords:** manipulação ortopédica, pé torto equinovaro congênito/cirurgia, pé torto equinovaro congênito/diagnóstico, pé torto equinovaro congênito/etiologia, procedimentos ortopédicos

## Abstract

O pé torto congênito é uma deformidade complexa, frequente e que pode ser desafiadora mesmo em mãos experientes. O método de Ponseti continua sendo universalmente aceito como padrão ouro para o tratamento e ótimos resultados são esperados na maioria dos casos com o manejo adequado da técnica. As recidivas continuam sendo um problema e estão associadas principalmente à não adesão ao uso da órtese. No entanto, outros fatores podem influenciar o risco de recidiva e contribuir para um resultado insatisfatório. A transferência do tibial anterior equilibra as forças deformantes e ajuda na correção, desde que o pé seja flexível. A deformidade recidivada não será corrigida espontaneamente, requer tratamento. Procedimentos cirúrgicos adjuvantes devem fazer parte do arsenal terapêutico do ortopedista.

## Introdução

O pé torto congênito (PTC) é uma das alterações músculo-esqueléticas congênitas mais frequentes. É caracterizado pela rigidez da deformidade com componentes em equino, varo, cavo e aduto. Apresenta incidência variando de 0.39 a 7 para cada mil nascidos vivos, de acordo com a etnia, sendo mais comum no sexo masculino e bilateral em 50% dos casos. 1 2

A etiologia é multifatorial e sua forma mais comum é a idiopática. Contudo, o PTC é também frequentemente associado a doenças neuromusculares, como mielomeningocele, artrogripose e diversas síndromes. 1 2 3

São considerados fatores de risco o tabagismo materno, diabetes gestacional, idade materna e infecções virais. Muitos genes têm sido associados ao PTC idiopático, com componente hereditário de até 20%. 3

A presença de rigidez é o que difere o PTC das deformidades posturais. Isso é facilmente identificado já no primeiro exame físico, quando não é possível a correção completa com a mobilização do pé.

Apesar do PTC se apresentar dentro de um espectro de maior ou menor gravidade, as seguintes alterações estão presentes: contratura do tendão calcâneo, encurtamento dos ligamentos deltoide e mola; encurtamento do tendão tibial posterior e tensão das estruturas capsuloligamentares posteromediais. Essas alterações levam ao desvio medial do navicular e do cuboide e ao varo da subtalar, gerando uma deformidade em inversão, adução e consequente supinação. O cavo é secundário à flexão do primeiro raio.

O tratamento ideal deve corrigir essas deformidades levando a um pé plantígrado, flexível, funcional e indolor. 1 2 O objetivo deste artigo é revisar os conceitos atuais na abordagem do PTC idiopático.

## Histórico

Os primeiros registros remetem ao antigo Egito no século XII a.C.. Em 400 a.C., Hipócrates descreveu o método de manipulação e imobilização para tratamento da deformidade, seguido de uso de sapatos especiais para manter a correção. No século XVIII foi introduzida a tenotomia. Novas técnicas cirúrgicas surgiram no século XIX com a introdução da anestesia e anti-sepsia. 1 2

A partir de então, as cirurgias com extensa liberação de partes moles, como as descritas por Turco, McKay e Simons, prevaleceram. No entanto, concomitantemente, os tratamentos menos invasivos foram ganhando força.

Kite, em 1939, descreveu sua técnica com manipulação e gesso seriado. Porém o fulcro de correção na calcâneo-cuboide limitava a correção do varo e exigia liberação de partes moles em 50 a 75% dos casos. 1 2

Na década seguinte à publicação de Kite, Ponseti desenvolveu o seu método na escola de medicina da Universidade de Iowa. Estudando as várias técnicas de correção do PTC, observou que a maioria evoluía com necessidade de abordagens cirúrgicas e consequente rigidez, fraqueza e extensas cicatrizes.

Observando Kite, na época o principal defensor do tratamento conservador, Ponseti conclui que a falha estava relacionada a uma compreensão equivocada da anatomia e da biomecânica no PTC. Concluiu que ao usar o fulcro no tálus e colocar pressão no primeiro metatarso, supinando o pé, era possível destravar a subtalar, etapa essencial como primeiro passo da manipulação. Este foi o início de um método revolucionário. Em 1963, publicou seus primeiros resultados a longo prazo, e atualmente a técnica de Ponseti é considerada o padrão ouro para o tratamento do PTC ( Fig. 1 ). 1 2

**Fig. 1:** ( **A** ) Recém-nascido com pé torto congênito (PTC) bilateral. ( **B** ) Criança com PTC unilateral à direita, aos 3 anos de idade, em uso da órtese de abdução (Denis Browne) por 14 horas/dia. Note como o pé acometido (direito) deve ser mantido com 70° de abdução enquanto o pé esquerdo é mantido a 40°. **(C)** Paciente aos 7 anos de idade, resultado do tratamento pela técnica de Ponseti para PTC à direita.

## Avaliação e classificação

O PTC pode ser classificado em quatro tipos: postural, idiopático, neurogênico e sindrômico. O pé torto postural é flexível e evolui com correção espontânea. A mobilização pode ajudar a se obter uma correção mais rápida. Caso necessite de manipulação e imobilização, provavelmente trata-se de um PTC menos grave. 2

O PTC idiopático, como o nome diz, não tem causa conhecida. É caracterizado por rigidez e requer tratamento com manipulação e imobilização gessada. 2 4 A tenotomia do tendão calcâneo é necessária na maioria dos casos e a manutenção da correção com uso de órtese é fundamental para previnir recidiva.

Trata-se de um grupo amplo, com variadas formas de gravidade, caracterizadas por maior ou menor rigidez. Apesar do sucesso do método de Ponseti no tratamento da imensa maioria dos pés idiopáticos, alguns pés são mais resistentes. Por vezes, podem exigir abordagens cirúrgicas mais complexas e apresentarem mais recidivas. Acredita-se que exista componentes muscular e genético determinantes nesses pés mais resistentes. 4

Os tipos sindrômicos e neurogênicos também podem responder ao método de Ponseti, apesar de mais rígidos, resistentes e com maior índice de recidiva. 5

Diferentes sistemas de classificação foram desenvolvidos para avaliar a gravidade, prognóstico e evolução do tratamento do PTC. As duas classificações mais utilizadas são as de Pirani e Diméglio. 2 6 7 8 9

Diméglio classificou o PTC considerando quatro parâmetros principais: equino, varo, rotação e adução, cada um recebendo uma pontuação de um a quatro de acordo com a redutibilidade. Os parâmetros secundários, quando presentes, somam um ponto cada: prega posterior, prega medial, cavo e condição muscular (contratura ou hipertonia do tríceps sural ou tibial anterior e/ou fraqueza dos fibulares). Assim, cada pé recebe uma pontuação de 0 a 20 e são divididos em 4 tipos de acordo com a soma: leve, moderado, grave e muito grave. 6

Grau I (0–5 pontos): leve, flexível e benigno, similar ao pé torto postural. Diméglio sugere, inclusive, que seja excluído das estatísticas por falsear positivamente os resultados.
Grau II (5–10 pontos): moderado, redutível, mas parcialmente resistente.
Grau III (10–15 pontos): grave, resistente, mas parcialmente redutível. É o tipo mais comum segundo Diméglio.
Grau IV (15–20): muito grave. É um pé muito rígido, praticamente irredutível, um tipo de pé pseudoartrogripótico.

Pirani baseou sua classificação em seis parâmetros, sendo três relacionados ao retropé (prega posterior, palpação do calcâneo e redutibilidade do equino) e três relacionados ao mediopé (prega medial, cobertura do tálus e borda lateral do pé). Cada um destes itens recebe uma pontuação de 0 (sem anormalidade), 0,5 (anormal) a 1 (muito anormal), chegando ao total de até 6 pontos. É uma escala útil para a avaliar a evolução do tratamento. 2 9

Ambas as classificações têm valor para documentação, mas pouca utilidade prática. Apesar de boa reprodutibilidade, a utilidade para prever prognóstico e guiar tratamento é controversa. No entanto, estudos recentes sugerem que ambas têm potencial para ajudar a prever o número de gessos e assim contribuir para um melhor alinhamento de expectativas com a família. 2 9

## Tratamento

Uma vez feito o diagnóstico do PTC, a abordagem deve ser precoce. A consulta pré-natal é uma oportunidade de orientar os pais sobre a história natural, expectativas, prognóstico e estratégias de tratamento. Desde o primeiro contato deve-se enfatizar que a adesão da família durante todo processo é fundamental para que se obtenha o resultado desejado.

Ponseti sugeriu que o tratamento se inicie na primeira semana de vida para aproveitar a maior flexibilidade do recém-nascido. 2 No entanto, não se trata de uma urgência ortopédica. Zionts et al. 10 não encontraram diferenças no número de gessos, incidência de lesões cutâneas, adesão ao tratamento ou recidiva ao comparar a idade de início do tratamento em 176 pacientes (mediana de 4 semanas de vida). Sugerimos que seja avaliado o contexto do recém-nascido de forma individualizada, considerando outros fatores para determinar o início do tratamento. É razoável que se espere os primeiros dias de adaptação da família, a primeira consulta com o pediatra após a alta, estabelecimento da amamentação e confirmação de um ganho de peso efetivo. Uma vez que as condições estejam favoráveis, não se deve protelar o tratamento.

O método de Ponseti é a primeira escolha, inclusive nos casos de PTC não idiopático. 1 2 11 Consiste em manipulação e imobilização com gesso inguinopodálico semanalmente para alongamento das estruturas posteromediais com contraturas e correção gradativa das deformidades. É fundamental que o fulcro da manipulação seja na face lateral da cabeça do tálus, que se encontra palpável na região dorso lateral do mediopé ( Fig. 2 ). A primeira deformidade a ser corrigida é o cavo, com apoio no colo do primeiro metatarso e uma manobra de supinação do antepé, para colocá-lo em alinhamento adequado com o retropé. A percepção equivocada de que é necessária pronação para a correção do pé causa um aumento do cavo, levando a uma deformidade iatrogênica. 1 2

**Fig. 2:** Técnica de Ponseti: ( **A,B** ) detalhe da manipulação com fulcro na cabeça do tálus e manobra de abdução com o pé supinado. ( **C,D** ) exemplos do primeiro gesso para correção do cavo com o pé supinado.

A seguir, inicia-se progressivamente a correção do aduto, varo e equino através de uma manobra de abdução. A contrapressão do polegar na cabeça do tálus evita a sua rotação na pinça do tornozelo. O varo, a inversão e a adução do retropé são corrigidos simultaneamente, pois as articulações do tarso estão em estrita interdependência mecânica e não podem ser corrigidas isoladamente. 2

Um gesso bem moldado é fundamental para manter o pé na posição de correção e evitar o deslizamento do aparelho gessado. Deve se utilizar apenas uma fina camada de algodão e fazer manipulações suaves, sem forçar a correção. Evite pressão contínua sobre o tálus durante a confecção do gesso e molde bem sobre a tuberosidade posterior do calcâneo e ao redor dos maléolos. O calcâneo não deve ser tocado durante a manipulação ou confecção do gesso para não limitar a mobilidade da subtalar e prejudicar a correção do varo. A moldagem é um processo dinâmico, movimente constantemente os dedos para evitar pressão excessiva sobre qualquer saliência óssea e continue moldando até que o gesso endureça. 1 2 O uso de água aquecida acelera a secagem do gesso. Confeccione inicialmente a bota gessada cobrindo os dedos e a seguir faça o acabamento, expondo os dedos, mas mantendo um suporte plantar para os artelhos. Finalmente, o gesso deve ser estendido até a raiz da coxa, mantendo o joelho sempre com flexão de 90°. 2 12 Estender o joelho pode facilitar a passagem do gesso, especialmente em bebês menores, mas isso deve ser evitado para que não ocorra pressão na região poplítea após a flexão do joelho.

São realizadas, em média, quatro a seis trocas gessadas antes da correção do equino, última deformidade a ser corrigida. A tenotomia, necessária em cerca de 90% dos casos, deve ser realizada após alcançada correção das componentes em cavo, aduto e varo, com uma abdução do pé de pelo menos 50° em relação ao plano da tíbia ( Fig. 3 ). A tentativa de correção do equino com manipulação forçada pode levar a uma deformidade em “mata borrão”, pois, diferente dos ligamentos do tarso, que podem ser alongados, o tendão do calcâneo é formado por fibras de colágeno não distensíveis, espessas e com poucas células. 2 Entretanto, em alguns casos, o ganho de dorsiflexão ocorre simultaneamente à correção das outras deformidades. Quando isso acontece e uma dorsiflexão de pelo menos 15° é observada, a tenotomia é desnecessária. 1 2 13

**Fig. 3:** ( **A,B** ) Pé torto congênito corrigido parcialmente após quatro gessos de Ponseti, mantendo limitação da dorsiflexão. ( **C,D** ) Resultado imediato após tenotomia percutânea com ganho satisfatório de dorsiflexão. ( **E,F** ) Detalhe do gesso pós tenotomia, mantendo a abdução e a dorsiflexão.

Ponseti preconiza a realização da tenotomia de forma percutânea e em ambiente ambulatorial, especialmente pelo menor custo, fácil reprodução e sem o risco anestésico. 2 14 No entanto, considerando o baixo risco da anestesia nos dias atuais, facilidade de realizar o procedimento com o paciente anestesiado, melhor manejo de eventual sangramento em um ambiente mais controlado, muitos cirurgiões optam pela realização do procedimento sob anestesia. 15 Deve-se ter o cuidado com o risco de uma tenotomia incompleta em casos de abordagem percutânea, levando a uma correção insatisfatória. A realização do procedimento sob visualização direta do tendão, através de um pequeno acesso, é recomendada especialmente em casos de nova tenotomia após recidiva. 13 Em pacientes com mielomeningocele recomenda-se a ressecção de cerca de 1 cm do tendão do calcâneo para diminuir o risco de recidiva, conforme demonstrado por Arkin et al. 5

O último gesso deve ser confeccionado com o pé em hipercorreção, cerca de 70° de abdução e 20° de dorsiflexão, e mantido por 3 semanas. A seguir, inicia-se o uso da órtese de abdução por 23 horas durante os 3 primeiros meses e depois por 14 a 16 horas por dia até os 4 anos de idade. 1 2 16

A órtese de abdução deve manter o pé corrigido em 70° de abdução e o pé não acometido, em caso de PTC unilateral, em 40° de abdução ( Fig. 1 ). A barra deve ter o comprimento igual à distância dos ombros e uma curvatura de cerca de 10° graus com a convexidade para baixo, ajudando a manter os pés em valgo e dorsiflexão. É fundamental manter o pé bem posicionado, totalmente apoiado, com as tiras e cadarços bem justos, para evitar que o calcâneo suba e o pé fique em equino.

A taxa de sucesso do tratamento do PTC idiopático pelo método de Ponseti é maior que 90%, desde que a técnica seja aplicada corretamente e a adesão ao uso da órtese seja mantida até a idade preconizada. 2 16 Uma preocupação frequente dos pais é se o tratamento prolongado trará algum atraso para o desenvolvimento da criança; porém, isso não ocorre de forma relevante. É esperado um atraso mínimo (1,5 mês) para atingir principais os marcos do desenvolvimento motor grosseiro e um atraso médio de 2 meses para a aquisição da marcha independente. 17 A habilidade para a prática de esportes também parece não ser prejudicada nas crianças em idade escolar que receberam tratamento adequado. 18

## PTC complexo

Descrito por Ponseti, o PTC complexo é considerado uma variação do PTC idiopático. É caracterizado por um equino rígido, flexão plantar severa dos metatarsos, prega profunda acima do calcanhar, prega transversal plantar e encurtamento do primeiro metatarso com hiperextensão do hálux ( Fig. 4 ). Essas características não são evidentes ao nascimento, se apresentam durante o tratamento e podem ter relação com técnica inadequada. Muitas vezes são identificadas após o deslizamento do gesso. 19 20

**Fig. 4:** ( **A** ) Exemplo de PTC complexo, caracterizado por rigidez, encurtamento do primeiro raio, extensão do hálux e prega transversa plantar. ( **B** ) Exemplo de PTC negligenciado aos 3 anos de idade.

O PTC complexo exige alguns cuidados no seu manejo e deve ser devidamente reconhecido. Embora a adução do antepé seja facilmente corrigida, os metatarsos permanecem em flexão plantar com o calcâneo fixo em equino e a tentativa de correção do restante das deformidades com persistência da manobra de abdução do antepé leva à hiperabdução dos metatarsos. Ponseti recomenda que a correção deve ser realizada com fulcro na cabeça do tálus e também no maléolo lateral. O antepé não deve ser abduzido além de seu alinhamento normal. Após a correção do varo, a flexão do antepé e o equino são corrigidos simultaneamente pela dorsiflexão dos metatarsos com ambos os polegares. No entanto, a tenotomia deve ser realizada para correção do equino residual, com cuidado para evitar a hiperextensão dos metatarsos e consequente progressão para deformidade em mata borrão. Para evitar que o gesso escorregue, o joelho deve ser imobilizado em pelo menos 110° de flexão. 2 19 20

## Recidiva

É considerada recidiva o retorno de qualquer componente do PTC (equino, cavo, varo, aduto e supinação) no pé já corrigido. A perda da dorsiflexão é geralmente a primeira alteração observada. Uma dorsiflexão de pelo menos 20° até três anos de idade, e de 15° até cinco anos, é esperada no pé corrigido. 21 22 Rieger e Dobbs consideram recidiva uma dorsiflexão menor que 15°. 1 Dietz sugere que se inicie o tratamento da recidiva em paciente com menos de 10° de dorsiflexão. 22 No caso de PTC unilateral, é esperada uma diferença de 10° entre os pés, um aumento nessa diferença pode ser um indicativo de recidiva. 22

A deformidade recidivada não será corrigida espontaneamente, requer tratamento. Geralmente um curto período de manipulação e gesso semanal, seguido da retomada do uso da órtese, é suficiente. Uma nova tenotomia pode ser necessária para se obter dorsiflexão satisfatória. 2

A supinação pode ser observada após o início da marcha, com varo e supinação dinâmicos, com apoio na borda lateral e espessamento da pele plantar lateral. Isso ocorre devido ao desequilíbrio entre o músculo tibial anterior forte e os músculos fibulares fracos. Nesses casos, a transferência do tendão tibial anterior (TTTA) pode ser necessária. Geralmente é indicada a partir de 3 anos de idade, após ossificação do cuneiforme lateral. A TTTA deve ser realizada somente quando a deformidade é dinâmica e o pé flexível, ou seja, é corrigível completamente com a mobilização passiva. Qualquer deformidade rígida deve ser corrigida por trocas gessadas, eventual tenotomia do tendão calcâneo para correção do equino, ou mesmo osteotomias, quando necessárias, antes da realização da TTTA. 2 13

É importante ressaltar que a causa mais comum de recidiva é a não aderência ao uso da órtese. A recorrência é cerca de 10 vezes maior quando isso ocorre. Segundo Desai et al., as recidivas ocorrem em apenas 6% quando se segue adequadamente o protocolo e em mais de 80% quando não há adesão. 1 2 16 Zionts et al. encontraram um risco de 42% de necessidade de TTTA em pacientes que não seguiram o protocolo de uso da órtese contra 19% em pacientes que usaram corretamente. 23

A adesão à órtese é o melhor preditor de recidiva do que a gravidade da deformidade ao nascimento. 1 16 No entanto, a recorrência ainda ocorre apesar do uso correto da órtese, sugerindo que existem outros componentes envolvidos que não foram totalmente elucidados. Embora a etiologia seja multifatorial e ainda não completamente esclarecida, há cada vez mais evidências apontando para uma etiologia neuromuscular e consequentes forças deformantes que persistem após a correção inicial. 2 23 24

Quando a recidiva ocorre mesmo com o uso correto da órtese, é provável que haja um componente neuromuscular mais relevante, com maior fraqueza de fibulares. A TTTA corrige esse desequilíbrio e melhora os parâmetros biomecânicos, tornando desnecessário, na maioria dos casos, o uso da órtese de abdução após o procedimento. 13 23 24 25 Little et al., em um estudo com 104 pacientes (172 pés), mostraram que a fraqueza dos eversores é importante causa de recidiva. Nos pacientes com fraqueza, 67,9% tiveram recidiva e 50% necessitaram de cirurgia adicional, por outro lado, nenhum dos pacientes que apresentavam boa força eversora tiveram recorrência. 24

## Pé torto após o início da marcha

É considerado um PTC negligenciado aquele sem tratamento prévio em pacientes após o início da marcha. ( Fig. 4 ). Deve-se diferenciar o PTC negligenciado do PTC residual, aquele que não foi completamente corrigido, e do PTC recidivado. Independentemente da idade ou gravidade, o método de Ponseti deve ser a escolha para o tratamento do PTC negligenciado, residual ou recidivado. 26 27

No PTC negligenciado, o resultado com o método de Ponseti pode ser surpreendente mesmo em pacientes mais velhos. No entanto, um maior número de trocas gessadas é necessário e há maior probabilidade de cirurgia complementar. 13 26 27 Lourenço e Morcuende 26 recomendam que esses pés sejam manipulados por 5 a 10 minutos antes da imobilização gessada e que o gesso seja mantido por duas semanas para maior ganho de flexibilidade.

Mesmo nos casos de recidiva tardia ou deformidade residual nos quais o pé se mostra resistente, trocas gessadas pré-operatórias contribuem para ganho de flexibilidade e podem trazer algum grau de correção, diminuindo a extensão da intervenção cirúrgica. 13 26 27

Inúmeras técnicas foram descritas para o tratamento do PTC, mas atualmente devem ser consideradas procedimentos adjuvantes em caso de falha da correção pelo método de Ponseti 4 13 ( Fig. 5 ).

**Fig. 5:** ( **A** ) Recém-nascido com PTC associado a artrogripose, aspecto inicial. ( **B** ) Após 12 semanas de manipulação pela técnica de Ponseti os pés se mantinham rígidos e resistentes. **(C e D)** Resultado aos 2 anos de idade, após liberação posteromedial lateral. ( **E–G** ) Detalhes da técnica de liberação radical pela incisão de Cincinnati; ( **E** ) isolados o feixe neurovascular e os tendões tibial posterior, flexor longo dos dedos e flexor longo do hálux. ( **F** ) Manobra de derrotação do tálus, conforme descrita por Hsu et al. 29 ( **G** ) Resultado após fixação das articulações subtalar e talonavicular.

As principais cirurgias podem ser divididas entre procedimentos de partes moles e ósseos. Historicamente, as técnicas de liberação ampla, posteromedial ou posteromédial lateral, são eficazes para correção das deformidades e obtenção de um pé plantígrado. Entretanto, levam à fibrose, retração cicatricial e consequente rigidez, com resultados insatisfatórios a longo prazo. 28 29 As liberações amplas devem ser reservadas, preferencialmente, para casos não idiopáticos. Quando indicada como tratamento adjuvante ao método de Ponseti, deve ser realizada sob medida, com a menor abordagem possível, visando corrigir apenas deformidades que não responderam às manipulações e trocas gessadas. 13

Se não houver melhora do equino após a tenotomia, deve-se realizar a liberação posterior (capsulotomia da tibiotársica e subtalar). Nesse caso, recomenda-se a dissecção cuidadosa para identificar os tendões fibulares, flexor longo do hálux, flexor longo dos dedos e o feixe neurovascular ( Fig. 5 ). Uma vez identificadas as estruturas, realiza-se a capsulotomia posterior, começando lateralmente, da bainha dos tendões fibulares, e indo medialmente até a bainha do flexor longo do hálux. 13

Caso haja necessidade, a liberação posterior pode ser estendida medial e/ou lateral, podendo chegar até a liberação peritalar completa, mas com as desvantagens e complicações já descritas. O deltoide profundo sempre deve ser preservado para evitar a hipercorreção em valgo, mas a ressecção do ligamento interósseo talocalcâneo é controversa. 13 30

Nos casos em que for necessária a liberação posteromedial lateral, Hsu et al. preconizam a derrotação do tálus com auxílio de um fio de Kirschner atuando como joystick para otimizar a correção antes da fixação talonavicular e subtalar 29 ( Fig. 5 ).

Outros procedimentos, como a capsulotomia calcaneocuboide, fasciotomia, liberações das cápsulas plantares das articulações da coluna medial, alongamento dos flexores dos dedos, do hálux, do tibial posterior e tenotomia do abdutor do hálux, podem ser necessários.

Os procedimentos ósseos são reservados para pacientes mais velhos com deformidades não redutíveis e estruturadas. A correção do varo do calcanhar pode ser feita pela técnica de Dwyer (osteotomia de ressecção de cunha lateral). Uma cunha de abertura no cuneiforme medial pode ser associada a uma cunha de fechamento lateral no cuboide para otimizar a correção do aduto. A osteotomia com uma cunha de base dorsal no mediopé auxilia na correção do cavo. A hemiepifisiodese anterior da tíbia distal é uma opção em caso de deformidade em equino residual e refratária. Procedimentos de salvamento como a naviculectomia combinada com encurtamento calcâneo-cuboide lateral, conforme descrito por Mubarak, talectomia e artrodese tríplice devem ser reservadas para casos graves e em pacientes mais velhos, assim como o uso do fixador externo circular com distração gradual e correção em múltiplos planos. 4 13

## Considerações finais

O método de Ponseti continua sendo o padrão ouro no tratamento do PTC. O pé complexo deve ser devidamente identificado pois o seu manejo adequado exige mudança na técnica. A recidiva continua sendo um problema e a sua principal causa é a não adesão ao protocolo de uso da órtese de abdução. A transferência do tibial anterior equilibra as forças deformantes no pé recidivado e com supinação dinâmica, desde que seja flexível. A deformidade residual ou recidivada não será corrigida espontaneamente e nenhuma transferência de tendão é capaz de corrigir um pé que não seja passivamente corrigível. Procedimentos cirúrgicos adjuvantes devem fazer parte do arsenal terapêutico do ortopedista que se propõe a tratar o PTC.
